# Evaluating changes in electronic gambling machine policy on user losses in an Australian jurisdiction

**DOI:** 10.1186/s12889-019-6814-1

**Published:** 2019-05-06

**Authors:** Matthew Stevens, Charles Livingstone

**Affiliations:** 1 Menzies School of Health Research, Charles Darwin University, PO Box 41096, Casuarina, NT 0811 Australia; 2 School of Public Health & Preventive Medicine, Monash University, 553 St Kilda Road, Melbourne, Vic 3004 Australia

**Keywords:** Electronic gambling machines, Pokies, Policy, Public health, Regulation, Problem gambling risk, Harm

## Abstract

**Background:**

Electronic gambling machines (EGMs) are in casinos and community venues (hotels and clubs) in all jurisdictions in Australia, except Western Australia (only in casino). EGMs have a range of features that can affect how people gamble, which can influence losses incurred by users. The Northern Territory Government recently changed two EGM policies – the introduction of note acceptors on EGMs in community venues, and an increase in the cap from 10 to 20 EGMs in hotels and 45 to 55 in clubs. This study evaluates two changes in EGM policy on user losses in community venues, and tracks changes in user losses per adult, EGM gambler, and EGM problem/moderate risk gambler between 2005 and 2015.

**Methods:**

Trends in venue numbers, EGM numbers, user losses and user losses per EGM by venue type and size are presented to determine if EGM policy changes affected user losses. Data from the 2005 and 2015 NT gambling surveys are used to determine EGM user losses per adult, per EGM gambler, and per EGM problem and/or moderate risk gambler, with several assumptions applied.

**Results:**

From 2010 (post smoking ban) to 2013 real user losses were stagnant, but from 2013 to 2017, real user losses in community venues increased 19, 9, 8 and 5% per annum, with increases higher in clubs and hotels with the maximum allowable number of EGMs. Over the same period user losses in the two casinos declined by 13%. Between 2005 and 2015, estimated user losses per EGM problem/moderate risk and problem gambler increased by 5 and 34% respectively.

**Conclusions:**

The analysis demonstrates that reductions in how much money gamblers can insert into an EGM (load-up limit), and/or the abolition of note acceptors, and reductions in the number of EGMs in venues is likely to reduce harm from EGM use. Given the demonstrated inability for Australian jurisdictions to identify and implement effective harm prevention and minimisation interventions, a national approach to gambling regulation in Australia may be desirable. Similarly, national co-ordination of research, particularly on EGMs and online betting is required to better understand changes in gambling policy on related harms.

## Introduction

Electronic gambling machines (EGMs) are a ubiquitous feature in community venues (hotels and clubs) across all jurisdictions in Australia, except Western Australia. Hotels, also known as pubs in Australia, are commercial for-profit businesses, while clubs are not-for-profit incorporated associations, and usually attached to a sporting club or clubs. EGMs are also located in the thirteen casinos spread across all jurisdictions, with Queensland housing four, two each in Tasmania and the Northern Territory (NT), and one in each of the other jurisdictions. As a form of gambling, EGMs have long been known to be the gambling product most associated with problem gambling risk and associated harms in Australia [1, 2]. The higher risk for EGM gambling is linked to a range of features including the rapid or continuous speed or ‘event frequency’ at which users can gamble [3, 4], and other structural characteristics including ‘near misses’ and ‘losses disguised as wins’ (LDWs) [5–7], and in their accessibility in community venues [8–11]. Interestingly, the link between LDWs, heightened arousal and more frequent gambling was established as far back as the 1980s [12].

Before going in to regulatory approaches that can influence harms associated with EGMs, it is worth highlighting changes to the International Classification of Diseases for Mortality and Morbidity Statistics that place gambling disorder in mortality and morbidity statistics [13]. The World Health Organisation (WHO) only recently included Gambling Disorder in the International Classification of Diseases (ICD-11) under 06 Mental, behavioural or neurodevelopmental disorders, 06C Disorders due to addictive behaviours, 06C50 Gambling disorder. This addition to the ICD coding system brings it more into line with definitions in the Diagnostic and Statistical Manual of Mental Disorders (DSM-5), and places gambling alongside Gaming disorder and acknowledges that it can contribute to mortality and morbidity [14]. The inclusion of gambling (and gaming) in the health statistics framework is welcome, and comes behind a growing evidence base that problem and moderate risk gambling contributes to the burden of disease in Australia and New Zealand at similar levels to severe and moderate alcohol disorder [15]. The slowness of government health departments to allocate resources to gambling harm is far from ideal, given the ubiquitousness of gambling products in Australia, and particularly EGMs, given they are currently the riskiest form of gambling available in Australia [1, 2].

EGMs must conform to a set of national standards for Australia and New Zealand, but each jurisdiction can apply different guidelines around parameter settings on the EGMs, such as return to user ratio, maximum bet per spin, near misses, LDWs, and how much money can be loaded into the EGM (the ‘load-up limit’) and in what denomination of notes or coins [16]. However, these jurisdictional differences are not well publicised. Such regulatory differences can affect gamblers’ style and risk of gambling harm [5, 6, 16, 17]. For example, Leino et al. [6] found that LDWs increased the odds that a gambler will continue to gamble, compared with a loss, but that this effect was less than the likelihood of continuing to gamble after a win. In Queensland and Tasmania, EGMs are not permitted to reward LDWs via reinforcement effects such as ‘winning’ sounds or messages. This is not the case in other jurisdictions. Other features of EGMs such as maximum bet per spin, the load-up limit, and the denomination of notes accepted may also affect user losses, and varies across jurisdictions in Australia.

For example, in New South Wales (NSW), EGM gamblers can load up to $7500 (though this was reduced to $5000 just prior to manuscript submission) into an EGM at one time, while in the Northern Territory (NT) up until December 2013, note acceptors were not allowed on EGMs in community venues (hotels and clubs), and gamblers loaded $1 coins into the machine, with a maximum amount of $250. However, in May 2013 the NT regulation was changed, with no consultation with either community, counselling services or academics. EGMs in community venues were subsequently modified for a load up limit of $1000 in any note denomination. The reasoning behind the change made by the NT Government was not clear, and goes against the latest evidence base, with a recent systematic review in Canada finding that removal of large note acceptors from EGMs to be one of the most effective strategies to reduce consumer harm associated with this gambling product [5], and the recommendation of a maximum $20 load-up by Australia’s Productivity Commission [2].

Additionally, the previous caps on numbers of machines in community venues were lifted in early 2015 from 10 to 20 for hotels and 45 to 55 for clubs. Venues were required to complete a social impact assessment to demonstrate that the introduction of new machines would not cause additional harm to the surrounding communities. All applications for increases in EGM numbers by community venues were approved leading to increased EGM numbers from December 2015. This fortuitously occurred just after the 2015 NT Gambling Prevalence and Wellbeing Survey was completed in the field [18]. The NT Government has now commissioned a repeat of the survey, to be conducted in the latter months of 2018. The presence of EGMs in community venues has been a politically contentious issue in Australia, with the two major political parties in the 2018 Tasmanian election having opposing views towards EGMs in community venues, with one major party’s policy to remove all EGMs from community venues. The 2005 NT Gambling Prevalence Survey found that 49% of NT adults support a decrease in poker machines in community venues, while the 2015 survey found 53 and 50% of adults endorsed a decrease in EGMs in clubs and hotels respectively, though this result was not available to government until after the policy change lifting the cap on community venue EGMs in early 2015 [18, 19].

Across Australia introduction of indoor smoking bans led to declines of between 5 and 10% in EGM user losses across all jurisdictions when introduced, with Victoria being the first to introduce bans and see reductions [20], and reductions observed across all jurisdictions [21]. Paradoxically, it was this policy that has led to the biggest reduction in EGM user losses, and likely the most successful in reducing rates of problem gambling. The EGM user losses from the NT’s two casinos provide an interesting comparison, and a natural policy experiment, as the casinos’ EGMs have always had note acceptors. Over this same period, user losses in the casinos dropped after the smoking ban and then user losses have remained stagnant (or decreased in real dollars) and did not show an increase from 2013, as was observed in community venue user losses [18]. The two most recent reports from the NT Director General report that community venue EGM user losses continued to grow after the introduction of note acceptors, followed by a doubling of EGMs permitted in hotels, and a 20% increase in clubs [22, 23].

Changes in EGM user loss can reflect policy changes, consumer preferences, or changes in accessibility to venues, and machines within venues. There were four changes to policy and regulation over the period 2003 to 2017 that have likely affected user losses and the number of EGMs operating in the NT over the last several years:

- Smoking ban in all venues started from 1 January 2010.
- Note acceptors allowed in community venues (hotels and clubs) from 28 May 2013, allowing users a maximum loading limit of $1000 using $20, $50 or $100 notes.
- Previous caps of 10 EGMs per hotel and 45 EGMs per club were lifted in July 2015 to allow hotels up to 20 EGMs and clubs up to 55 EGMs, though no new EGMs were installed until after social impact assessments were carried out and reviewed by the government, which occurred in December 2015 and early 2016).
- Minimum percentage return to player (RTP) was amended on 21 September 2015 for casinos from 88 to 85%, which brought them into line with community venues.

Interestingly, EGM data obtained from the NT Government and published in the 2015 Gambling Prevalence and Wellbeing Survey report showed that RTP in community venues has, on average, increased between 2003/4 and 2014/15 from 88.6 to 90.5% [18]. For casinos over the same period the RTP was between 91.1 and 91.9%. Thus, although the change in minimum RTP standardises casino and community venue minimum RTP, the change does not reflect venue (casino and community) practices regarding RTP.

The inconsistency in EGM regulation across Australia, and the lack of finely grained data, has limited public health researchers’ capacity to evaluate EGM regulatory changes. However, the recent change in EGM policy to allow the installation of note acceptors and an increase in number of EGMs in community venues in the NT is a policy change that warrants investigation, given research has shown that removal of large note acceptors from EGMs can lead to reductions in problematic gambling behaviour [24]. It provides an opportunity to assess changes in community venue user losses and compare over time (before and after note acceptors and increase in EGMs) with user losses from casino EGMs (which have always had note acceptors).

This paper will evaluate the effect of the installation of note acceptors and increased load amount in community venues in 2013, and the change in EGM numbers occurring in late 2015 on user losses (and user losses per EGM) by venue type (hotel, club and casino) and size (as measured by number of EGMs in the venue). The paper will also use data from the 2005 and 2015 NT Gambling Prevalence Surveys to estimate changes in user losses per adult (18 years or more), per EGM user and per EGM problem and/or moderate risk gambler as classified by the Problem Gambling Severity Index (PGSI).

## Methods

### Data sources

The NT Government Department of Attorney General and Justice provided two sets of EGM data for the research. The first included venue name, monthly user losses and number of EGMs for the years 2003 to 2017. The second data set was at the EGM level and included venue name, and the date corresponding to the first insertion of notes into the machine. This second data set was for the years 2013 to 2017, and was for the period following policy change allowing note acceptors into community venue EGMs.

The NT Gambling Prevalence and Wellbeing Survey was undertaken in late 2015 and was the follow-up survey to the 2005 NT Gambling Prevalence Survey. Full details for both survey designs are available in Stevens et al. [18] and Young et al. [19], with a summary provided here. The 2005 NT Gambling Prevalence Survey, conducted August to September, replicated the methods used in the Productivity Commission’s 1999 national survey, and used a two-stage population survey with a stratified (age, sex, region), quota-based random CATI telephone sample of adults in the NT. All respondents were screened for gambling (all types) and categorised as non-gamblers, regular and non-regular gamblers, with regular gamblers being screened for problem gambling risk. A sub-sample of these groups then received the full survey (all regular gamblers and one in four non-regular gamblers, and one in two non-gamblers). The sample frame included all households with a telephone number listed in the NT telephone directory, with the last birthday method used to recruit respondents. The response rates were determined using the conservative method and the upper bound method, both of which calculate the response rate based on number of respondents who participated as a proportion of those eligible to participate, with the latter also including calls where there were no replies, answering machines or engaged numbers, and gave response rates of 32 and 37% receptively.

The 2015 NT Gambling Prevalence and Wellbeing Survey used a similar two-stage population survey with a stratified (age, sex, region), quota-based random CATI telephone sample of adults in the NT. However, dual frame sampling was used with a landline frame and three separate mobile phone lists used to draw a random sample of mobile phone users, to capture adults who predominantly or only use a mobile phone [25]. The three mobile lists were merged and de-duplication steps undertaken, and from this list, mobile numbers were randomly sampled. The ‘last birthday’ approach was again used to select a respondent within the household for the landline sample, though about midway through sampling, it was noticed that females were being oversampled, so interviewers changed to asking to speak with the male in the house who had the last birthday. Stratified sampling using region (Darwin/Palmerston, Alice Springs, Katherine, Tennant Creek/Nhulunbuy and the Rest of NT), gender (male, female) and age (18–34, 35–49, 50–64 and 65 or more years), was used, with broad territory wide proportional quotas set for region, age and gender.

However, the regular gambler screener was not used in the 2015 survey because of the bias it introduces in problem gambling risk estimates [18, 26]. All people who gambled were administered the PGSI. Respondents were then categorised according to at-risk gamblers (problem, moderate and low risk), non-risk gamblers and non-gamblers, with all at-risk gamblers, and one in four non-risk and non-gamblers receiving the full survey. Some questions were also designed for EGM gamblers with these respondents filtered through additional EGM specific questions. Consistent with the 2005 survey, the 2015 survey was carried out in the latter part of the year. The overall response rate using the conservative and upper bound methods was 25% (37% mobile, 22% landline) and 31% (44% mobile and 28% landline) respectively. Both surveys were weighted to the adult estimated resident population of the NT using Australian Bureau of Statistics data, with the 2015 survey using separate population weights for the Aboriginal and Torres Strait Islander and non-Indigenous samples. Population weights were adjusted for the probability of selection based on the different proportions of at-risk gamblers, non-risk gamblers and non-gamblers completing the full survey, in addition to estimated probability of selection based on mobile or landline respondent selection.

### Key variables

*User loss* is the amount of money lost on EGMs, or the difference between how much the user puts in the EGM and how much they take home after finishing the session. This is also referred to as Net Gambling Revenue (NGR). Conversely, from a venue point of view, user losses represent EGM revenues. *Number of EGMs* was measured per venue and is reported annually, and is the average number of operating EGMs in each month. *User losses per EGM* was derived by dividing the user losses for a venue by the number of operating EGMs for that venue for each year and provides a measure of playing intensity for that venue (type). *Venue size* is measured as the average number of operating EGMs in a venue for the year, with hotels divided into (i) less than 10 EGMs, (ii) 10 to 20 EGMs and clubs divided into (i) less than 20 EGMs, (ii) 20 to 44, and (iii) 45 to 55. Hotels were categorised based on whether they had the maximum allowable EGMs in the venue (i.e. old cap of 10), while for clubs the split was based on the previous cap of 45 and the need to observe differences in the uptake of note acceptors and changes in EGM in user losses for venues of varying size. So, the upper category for hotels and clubs includes venues with the maximum allowable number of EGMs under the previous cap, and venues at the old cap that increased EGM numbers to the new cap, once the cap was increased. *Problem gambling* risk was measured using the 9-item PGSI [27] using the original Likert scale, and standard categories of low risk (scores 1 or 2), moderate risk, (scores 3 to 7), and problem gambling (scores 8 or higher). *Weighted estimated resident populations* from the 2005 and 2015 surveys were used to derive EGM user losses per NT adult, per EGM user, per EGM problem/moderate risk gambler and per EGM problem gambler.

### Data management and analysis

EGM level data contained information on the venue name, EGM ID and date when notes were first inserted in to the EGM, which was aggregated to annual data giving venue by year data for number of new EGMs with note acceptors installed. This data was then merged with the user loss and number of EGMs data set by venue and year. Annual (calendar) time series trends for user losses, number of EGMs (by note acceptor status) and user losses per EGM were calculated by venue size. Descriptive statistics on the number of EGMs by venue size are shown for 2013 to 2017; the period of policy change for note acceptors and increased caps. Visual inspection and percentage change is used to describe annual changes over time in relation to changes in EGM policy (note acceptors and number of new EGMs). All EGM user losses are presented in ‘real’ dollar values, adjusted to 2017 dollar values using the Darwin Consumer Price Index (CPI) [28].

EGM user losses per adult for 2005 and 2015 are calculated for the total NT adult population, the EGM gambling population, and EGM problem/moderate risk gamblers, and percentage change calculated. Using the adult population is appropriate as EGMs are illegal for people under the age of 18 years and are EGMs located in venues licenced to sell alcohol. The 2005 survey estimates of problem and moderate risk gambling amongst EGM gamblers were adjusted to be comparable with the 2015 data because the 2005 survey only screened regular gamblers (weekly gamblers excluding lotto and instant scratch ticket gamblers), which meant problem gambling risk was significantly underestimated in the 2005 survey [26]. An adjustment factor was derived by applying the regular gambler filter from the 2005 survey to the 2015 survey and calculating the percentage of problem/moderate risk gamblers underestimated because of the regular gambler filter – this proportion was then applied to the 2015 EGM gamblers to obtain unbiased problem gambling risk estimates for EGM gamblers. To estimate user losses per adult, EGM gambler, per EGM problem gambler, and per EGM problem and problem/moderate risk gambler, an assumption needed to be made on what percentage of EGM user losses were gambled by non-NT residents. No information was available for this, so three possibilities were used: (i) 10% of user losses are from non-NT residents, (ii) 20% of user losses are from non-NT residents, and (iii) 30% of user losses are from non-NT residents. The Productivity Commission estimate [2] of 40% was used for the share of EGM user losses attributable to EGM problem gamblers; while the estimate from the 2005 NT Gambling Prevalence Survey [19] was used for the share attributable to EGM problem/moderate risk gamblers, which was 55%. The estimate used for problem gamblers was considered conservative by the Productivity Commission, while the 2005 estimate is the best available, as EGM user spend was not collected in the 2015 NT Gambling Prevalence and Wellbeing Survey. For this analysis, EGM user losses are presented as ‘real’ dollar values, based on the 2015 values.

Ethics approval was received to conduct and report on the 2015 Northern Territory Gambling Prevalence and Wellbeing Survey from the joint Northern Territory Department of Health and Menzies School of Health Research Humans Research Ethics Committee (2015–2369). Ethics approval was obtained for the 2005 Gambling Prevalence Survey from Charles Darwin University Human Research Ethics Committee. Verbal consent was obtained from all participants in both of these surveys, rather than written consent, as they were population prevalence surveys conducted using Computer Aided Telephone Interviewing (CATI). Approval to use EGM data was made by the Director General of Licensing, NT Department of Attorney General and Justice, and the analysis of EGM data constitutes secondary data analysis of administrative data (and this data contains no information on individuals).

## Results

**Fig. 1:**
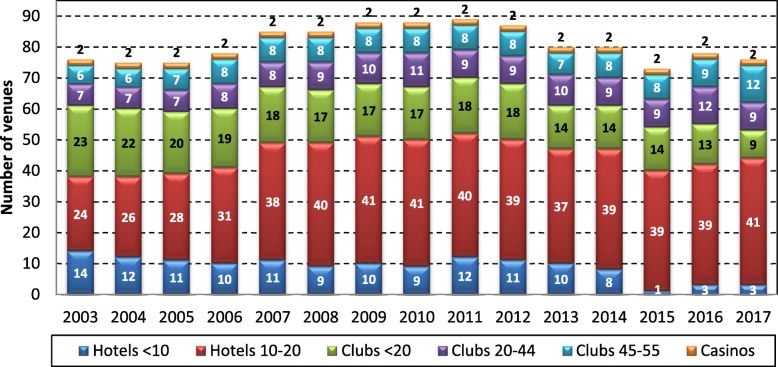
Number of community venues by venue type and size in the NT, 2003 to 2017

Figure 1 shows that the number of community venues (hotels and clubs) in the NT ranged between 71 and 87 over the period from 2003 to 2017, peaking at 87 in 2011, before declining again to 74 in 2017. The number of casinos operating EGMs in the NT was constant at two from 2003 to 2017. The number of hotels with EGMs increased from 38 in 2003 to 52 in 2011, and then declined to 40 in 2015, and increased again to 44 in 2017. The number of clubs with EGMs has been ranged between 34 and 36 between 2003 and 2012, and then declined from 35 in 2012 to 30 in 2017. The lifting of the EGM cap in 2015 is evident, with less hotels and clubs in the categories of venue size below the old EGM cap of 10 EGMs per hotel and 45 EGMs per club.

Table 1 shows changes in venue size and descriptive statistics on number of operating EGMs by venue size. Nine out of thirteen hotels that housed less than 10 EGMs in 2013, moved to housing 10 to 20 in 2017. Of the 39 hotels with 10 to 20 EGMs in 2017, sixteen venues had the new maximum cap of 20 EGMs, while 21 still contained 10 EGMs, with an average of 14 EGMs per hotels with 10 to 20 EGMs at the end of 2017. Of the 17 clubs that had less than 20 EGMs in 2013, one moved up to house 20 to 44 EGMs, while 2 jumped up to the 45 to 55 EGM size, while the average number of EGMs in clubs with less than 20 was seven in 2017. Of the nine clubs that housed 20 to 44 EGMs in 2013, five moved up a size category to 45 to 55 EGMs, while the average number of EGMs for venues with 20–44 EGMs was 29 in 2017. Of the five clubs that housed 45 EGMs in 2013, all remained in this category, with a mean number of EGMs in clubs with 45 to 55 EGMs being 50, with five housing 45 EGMs and 6 housing 55 EGMs. The average number of EGMs by venue size at the start of 2013 reflects that many venues were at the previous cap (10 for hotels and 45 for clubs).

**Table 1:** Number of venues by venue size at start of 2013 and end of 2017

	Venue size end of 2017								
Venue Size end of 2013	Hotel: <10	Hotel: 10-20	Club: <20	Club: 20-44	Club: 45-55	Total	Min EGMs	Max EGMs	Mean EGMs
Hotel: <10	5^a^	9				14	0	9	6
Hotel: 10-20	2^b^	30				32	10	10	10
Club: <20			13^c^	2	1	16	0	19	10
Club: 20-44			1	4	5	10	22	44	31
Club: 45-55					5	5	45	45	45
Total	7	39	14	6	11	77	-	-	-
Min EGMs	0	10	0	20	45	-	-	-	-
Max EGMs	8	20	17	44	55	-	-	-	-
Mean EGMs	3	14	7	29	50				

^a^Includes two hotels that now have no EGMs and two hotels that closed; ^b^Includes two hotels that closed between 2013 and 2017; ^c^Includes three clubs that now have no EGMs and two clubs that closed

**Fig. 2:**
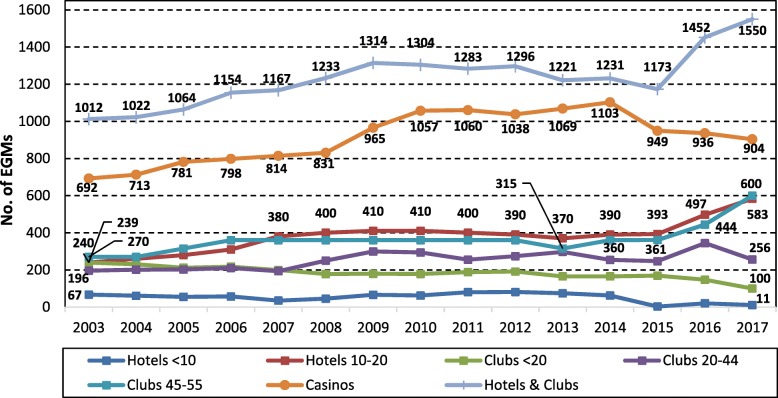
Number of EGMs by community venue type and size in the NT, 2003 to 2017

Figure 2 shows trends in number of EGMs by venue type and size. In 2003 there were 1704 EGMs in venues across the NT with 41% (705) in clubs, 18% (307) in hotels and 41% (692) in the two casinos. The number of EGMs in the two casinos increased between 2003 and 2014 from 692 to 1103, and then declined through to 2017, at which time they housed 904 EGMs. Trends for EGM numbers in community venues (hotels and clubs) followed a similar trend to numbers in the casinos, except that from 2015 onwards numbers increased 32% from 1173 to 1550 following the lifting of the caps. Most of the growth in new EGMs occurred in hotels and clubs that already had the maximum allowable number EGMs under the old cap, with a 66% increase in EGM numbers in clubs and 48% in hotels from 2015 to 2017.

**Fig. 3:**
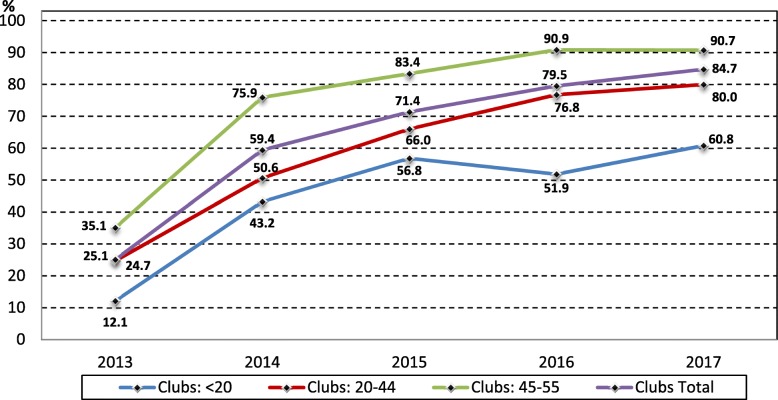
Number of EGMs by community venue type and size in the NT, 2003 to 2017

**Fig. 4:**
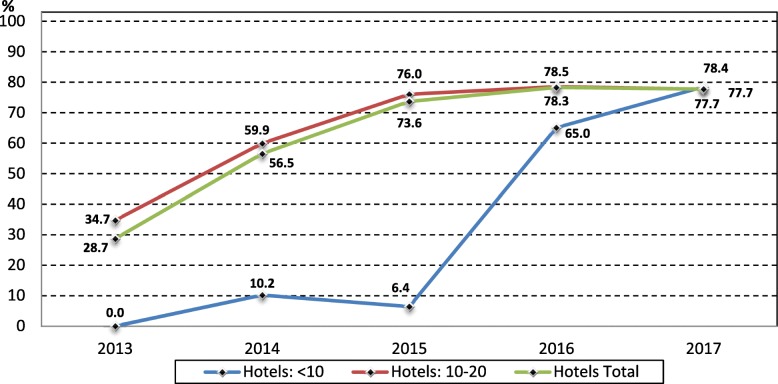
Number of EGMs by community venue type and size in the NT, 2003 to 2017

Figures 3 and 4 show the percentage of EGMs with note acceptors by venue size for clubs and hotels respectively. Casino EGMs are not shown as their EGMs have always had note acceptors (with the same loading limit). By the end of 2013 note acceptors were installed in 35% of EGMs in clubs with 45 to 55 EGMs, compared to 25% in clubs with 20 to 44 EGMs, and 12% in clubs with less than 20 EGMs. The percentage of EGMs with note acceptors in clubs with 45 or more EGMs rose sharply from 35% in 2013 to 76% in 2014, and then rose steadily from 2015 (83%) to 2017 (91%). The percentage of EGMs with note acceptors in clubs with 20 to 44 EGMs rose from 25% in 2013 to 80% in 2017 and is approaching the same as clubs with 45 to 55 EGMs. By the end of 2014 smaller clubs with less than 10 EGMs had installed note acceptors on 43% of EGMs, rising to 61% in 2017.

The percentage of EGMs with note acceptors in hotels reached 29% by the end of 2013 and rose to 78% by 2017. After the cap was lifted from 10 to 20 EGMs in hotels in 2015, most hotels had 10 or more EGMs, with only 11 EGMs located in hotels with less than 10 EGMs (Fig. 2), and most of these smaller hotels did not install note acceptors until 2016, when 65% of EGMs had note acceptors and this rose to 78% in 2017. The trend in percentage note acceptor EGMs for hotels with 10 to 20 EGMs was very like the total hotels and rose steadily from 35% in 2013 to 76% in 2015 before plateauing at around 78% in 2016 and 2017.

**Fig. 5:**
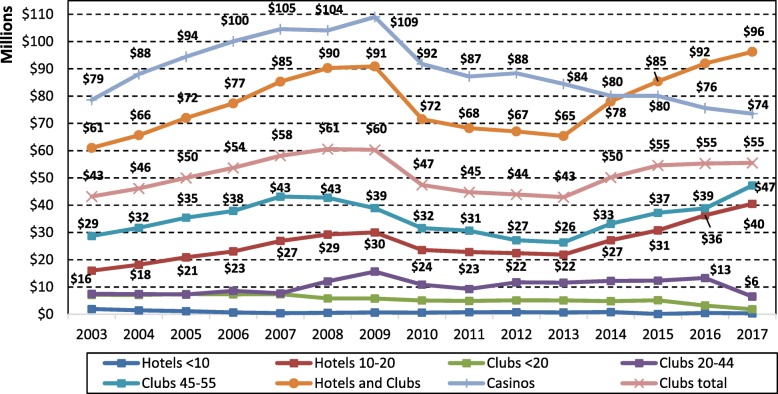
EGM user losses by community venue type and size in the NT, 2003 to 2017

Figure 5 shows real EGM user losses by venue type and size, with all dollar values adjusted to reflect 2017 dollar values using the Darwin CPI [28]. Total user losses in community venues increased dramatically after the change in policy in 2013 allowing note acceptors to be installed, and increased 19% from $65 million in 2013 to $78 million in 2014, and continued increasing to $96 million in 2017. This was a 47% increase in user losses over four years, that followed 4 years decreases in user losses from 2010 (first year of smoking ban) to 2013. In 2015, user losses in community venues in the NT surpassed user losses from EGMs in casinos for the first time. Over the same period in which note acceptors were introduced into community venues, casino user losses dropped from $80 million in 2013 to $74 million in 2017, with losses in the casinos declining since the smoking ban in 2010. EGM user losses only increased substantially in hotels and clubs with the maximum allowable EGMs, and it was also in these larger venues that note acceptors were installed more rapidly compared with smaller venues (Figs. 3 and 4). EGM user losses for all hotels is not shown as the user losses occurring in hotels with less than ten EGMs was negligible over time, due to fewer hotels having less than ten EGMs and the small number of EGMs housed in these hotels. EGM user losses in hotels increased by 82% between 2013 and 2017, with most of this increase occurring after the introduction of note acceptors, though the trajectory continued a similar path after more EGMs were added to venues after the lifting of the caps (note venue size changed over time, particularly after 2015 – see Table 1.

**Fig. 6:**
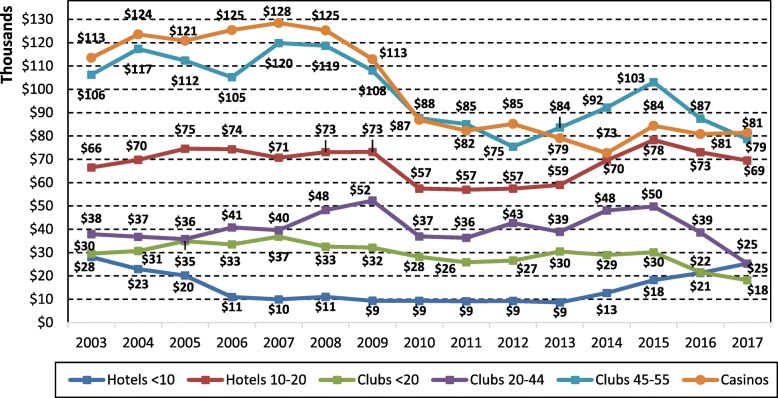
User losses per EGM by venue type and size in the NT, 2003 to 2017

Figure 6 plots real user losses per EGM by venue type and size. The trend for community venues user losses per EGM was virtually identical in trajectory to the hotels 10–20 and is not shown. After the smoking ban in 2010, user losses per EGM dropped in all venue types and size, though the drop was largest for the casinos, and clubs and hotels with the maximum allowable EGMs, though user losses per EGM had already began to decline in 2009 for clubs with 45 EGMs and the casinos. In hotels with less than 10 EGMs, user losses per EGM declined between 2003 to 2006, and then hovered around $9000 to $11,000 from 2006 to 2013, and then rose to $13,000 in 2014 after note acceptors could be installed, and continued to increase through to 2017 to $25,000. In contrast, user losses per EGM in hotels with 10 (to 20) EGMs increased from $66,000 to $73,000 between 2003 to 2009, before declining to $57,000 in 2010 (smoking ban), and then increasing slowly from 2010 to 2013 ($59,000), before a sharp increase of 24% to $70,000 in 2014 (after noter acceptors allowed) and $78,000 in 2015, and decreased in 2016 and 2017 to $69,000 after the lifting of the EGM cap from 10 to 20. User losses per EGM in clubs with less than 20 EGMs decreased from $30,000 in 2003 to $26,000 in 2011, and then increased slightly through to 2015 ($30,000), then decreased from 2015 to 2017 to $18,000 in the 2 years after EGM caps were increased. The trend line in user losses per EGM for clubs with 20 to 44 EGMs follows a similar trajectory to hotels with 10 to 20 EGMs, albeit with smaller user losses per EGM and climbs steadily from 2003 ($38,000) to 2009 ($52,000), before dropping after the smoking ban in 2010 ($37,000), remaining steady to 2013, before increasing to $50,000 in 2015, and then declining to $39,000 in 2016 and $25,000 in 2017.

### Share of top 10 user loss venues of total user losses

Table 2 shows that the user losses from the top 10 clubs were 81% of total user losses in all clubs, while these venues housed 62% of the EGMs in clubs. The average return per EGM ($88,086) was 30% higher in the top 10 clubs compared to all clubs ($67,849). The top 10 hotels share of all user losses in hotels was 58%, while the top 10 user loss hotels housed 35% of all EGMs in hotels. The average return per EGM ($117,834) in the top 10 hotels was 67% higher that the user loss per EGM across all hotels ($70,676). EGMs in hotels had higher user losses per EGM compared with clubs. Following the installation of note acceptors (2013–2015), the top 10 hotels had larger percentage increases in user losses (43%) compared with the top 10 clubs (18%), and a larger percentage increase in all hotels user losses (21%) compared with clubs (14%). Since the change in EGM policy in 2013, user losses have increased 30% in the top 10 clubs, compared with all clubs 26%, and user losses have increased 112% in the top 10 hotels, compared with 59% across all hotels.

**Table 2:** User losses, number of EGMs, user losses per EGM, change in real user losses and percentage share of top 10 venues for hotels and clubs, 2017

	2017			Real User losses		
	User losses $	Number of EGMs	User loss per EGM $	2013–2015% change	2015–2017% change	2013–2017% change
Top 10 clubs	$44,850,676	509	$88,086	17.9	10.1	29.8
Total clubs	$55,466,206	818	$67,849	14.2	10.2	25.9
% share top 10 clubs	81%	62%	–	–	–	–
Top 10 hotels	$23,488,255	199	$117,834	43.4	47.9	112.1
Total hotel	$40,768,368	577	$70,676	20.6	31.6	58.7
% share top 10 of hotels	58%	35%	–	–	–	–

### EGM gamblers and problem gambling risk

Table 3 shows annual, monthly and weekly prevalence of EGM gambling, with corresponding problem/moderate risk and problem gambling prevalence. There were decreases in annual, monthly and weekly participation in EGM gambling from 2005 to 2015. Just under a quarter of NT adults gambled on an EGM in the year prior to the 2015 survey, dropping to 2.7% for monthly use, and 1.4% for weekly use. In 2015, problem/moderate risk gambling prevalence among people who played EGMs was 10.5% and significantly higher compared with people who did not play EGMs (2.2%), but slightly lower than what was observed in the 2005 survey using adjusted prevalence. Problem/moderate risk gambling increased to 25% amongst monthly EGM gamblers, and further increased to 53% amongst weekly EGM gamblers in 2015. In 2005 and 2015 problem gambling prevalence amongst non-EGM gamblers was less than 0.5%, increasing to 4% (2005) and 2.7% (2015) amongst annual EGM gamblers. Around 8% (adjusted) of monthly EGM gamblers were classified as problem gamblers in 2005 and 14% in 2015, while 19% of weekly EGM gamblers were classified as problem gamblers in 2005 and 13% in 2015. Care should be made interpreting changes between surveys due to the methodological differences and the use of the adjustment factor for problem gambling risk.

**Table 3:** Population prevalence and problem and problem or moderate risk gambling prevalence by non-EGM gambling (but gambles), annual, monthly and weekly EGM gambling, 2005 and 2015

	Population prevalence		Problem/MR gambler^a^		Problem gambler^a^	
	% (SE)	Population (N)	% (SE)	Population (N)	% (SE)	Population (N)
2005 survey						
Non-EGM gambler^b^	73.0 (1.5)	100,918	0.7 (0.3)	733	0.02 (0.02)	24
EGM Gambler^c^	27.0 (1.5)	37,307	12.0 (1.6)	4492	4.0 (2.0)	1500
EGM 1–3 times per month	6.5 (0.8)	9019	19.4 (5.0)	1753	7.8 (1.5)	701
EGM Weekly or more	2.5 (0.3)	3391	44.1 (5.2)	1494	19.0 (3.8)	645
2015 survey						
Non-EGM gambler^b^	77.1 (1.3)	136,345	1.5 (0.5)	2067	0.07 (0.05)	95
EGM Gambler^c^	22.9 (1.3)	40,571	10.5 (1.8)	4268	2.7 (0.9)	1111
EGM 1–3 times per month	2.7 (0.4)	4784	24.8 (7.4)	1187	13.7 (6.4)	654
EGM Weekly or more	1.4 (0.3)	2498	52.8 (9.1)	1320	13.3 (6.3)	333

^a^ 2005 annual and monthly problem/moderate risk (MR) gambling estimates adjusted for bias caused by only sampling regular gamblers in the 2005 survey: 2015 proportion of monthly and less than monthly EGM gamblers that were not classified as problem/moderate risk gamblers was applied to the 2005 EGM participation data; ^b^ Includes everyone who gambled in the year before the survey, but did not gamble on EGMs; ^c^ Includes all people that gambled on an EGM once or more in the year before the survey

*Sources*: 2005 NT Gambling Prevalence Survey and 2015 NT Gambling Prevalence and Wellbeing Survey

### EGM user losses per person

Table 4 uses population data from the 2005 and 2015 surveys and combines it with EGM user loss data to estimate EGM user losses per NT adult, per EGM gambler, per EGM problem/moderate risk gambler and per EGM problem gambler. Darwin CPI adjusted (real) dollar values are presented for 2005 data. The following description refers to the real dollar values. Between 2005 ($165.6 million) and 2015 ($164.7 million) there was a − 0.6% decrease in real EGM user losses in the NT. EGM user losses per NT adult decreased from between $839 and $1078 to $652 and $838 depending on the 10, 20 and 30% assumption of EGM user losses to non-NT residents, which was a 22% decrease in EGM user losses per person. From 2005 to 2015 EGM real user losses per EGM gambler decreased 9%, equating to between $3108 and $3995 lost per EGM user in 2005 to between $2841 and $3653 loss per EGM user in 2015. Real losses amongst EGM problem/moderate risk gamblers increased 5% from 2005 to 2015. Depending on the assumptions used, in 2005 each EGM problem/moderate risk gambler lost on average between $10,323 and $18,249 per year (real), rising to between $10,804 and $19,100 in 2015. Lastly, real user losses per EGM problem gambler increased 34% and was between $30,915 and $39,748 in 2005 to between $41,504 and $53,362 in 2015.

**Table 4:** Real^a^ EGM user losses, losses per EGM user, estimate for losses per problem or moderate risk gambler, and user loss per NT adult, 2005 and 2015

	Real		
	2005	2015	% Change
EGM user losses ($)	$165,618,368	$164,682,559	−0.6
NT adult population (N)	138,225	176,916	28.0
User loss per NT adult ($)^c^	$1078	$838	−22.3
User loss per NT adult ($)^d^	$959	$745	−22.3
User loss per NT adult ($)^e^	$839	$652	−22.3
EGM population (N)	37,307	40,571	8.7
User loss per EGM gambler ($)^c^	$3995	$3653	−8.6
User loss per EGM gambler ($)^d^	$3551	$3247	−8.6
User loss per EGM gambler ($)^e^	$3108	$2841	−8.6
EGM problem/moderate risk gamblers (N)	4492	4268	−5.0
User loss per EGM PG/MR^b^ gambler ($)^c,f^	$13,272	$13,891	4.7
User loss per EGM PG/MR gambler ($)^c,g^	$18,249	$19,100	4.7
User loss per EGM PG/MR^¥^ gambler ($)^d,f^	$11,797	$12,347	4.7
User loss per EGM PG/MR gambler ($)^d,g^	$16,221	$16,978	4.7
User loss per EGM PG/MR gambler ($)^e,f^	$10,323	$10,804	4.7
User loss per EGM PG/MR gambler ($)^e,g^	$14,194	$14,855	4.7
EGM problem gamblers (N)	1500	1111	−25.9
User loss per EGM problem gambler ($)^c,f^	$39,748	$53,362	34.3
User loss per EGM problem gambler ($)^d,f^	$35,332	$47,433	34.3
User loss per EGM problem gambler ($)^e,f^	$30,915	$41,504	34.3

^a^ Darwin All groups Consumer Price Index 2015 dollars [28]

^b^ PG/MR = problem/moderate risk gambler

^c^ Assumes 10% of EGM user losses from non-NT residents

^d^ Assumes 20% of EGM user losses from non-NT residents

^e^ Assumes 30% of EGM user losses from non-NT residents

^f^ Assumes 40% of EGM user losses from problem(/moderate) gamblers (Source: 2)

^g^ Assumes 55% of EGM user losses from problem/moderate risk gamblers (from 2005 NT Gambling Prevalence Survey EGM expenditure data)

*Sources*: 2005 NT Gambling Prevalence Survey; 2015 NT Gambling Prevalence and Wellbeing Survey; NT Government EGM data

## Discussion

The analysis of EGM user losses showed that the introduction of note acceptors into community venues led to large increases in user losses in these venues, while over the same period, user losses from EGMs located in the two casinos remained flat or declined. The change in EGM policy in 2013 allowed note acceptors to be installed on EGMs located in community venues in the NT, but it also increased the maximum allowable amount to be loaded into an EGM at one time from $250 to $1000. This change brings EGM loading rules in NT community venues closer to other jurisdictions, but is inconsistent with the most recent Productivity Commission report [2], which recommended restricting the amount a user can load into a machine at any one time to $20. User losses from EGMs in community venues ($85 million) overtook user losses in the two casinos (combined $80 million) for the first time in 2015, and continued to increase to $96 million in 2017, while declining in the two casinos to $74 million. The increase in user losses predominantly occurred before the lifting of the cap on EGM numbers in community venues, which came into effect in 2015, but with only a few venues increasing their EGM numbers in 2015 due to the social impact assessment process required by the government. However, by the end of 2017 the percentage of the total EGMs housed in clubs (39%, 956 EGMs) was greater than the casinos (37%, 904 EGMs) for the first time since 2003. The decline in user losses in casino EGMs is likely to be partly attributable to the decrease in EGM numbers housed in the two casinos, but may also reflect EGM gamblers choosing to use EGMs in community venues after the introduction of note acceptors.

Since the lifting of the caps in community EGM venues in 2015, there has been a 50% increase in EGM numbers in hotels, and a 23% increase in clubs, while over the same period the two casinos had a 5% decrease in EGM numbers. Then in November 2017, the NT Government announced they were reinstalling a cap on EGM numbers, with the overall total cap set at 1734 EGMs, with the 20 and 55 EGMs cap applying to hotels and clubs respectively, which is 176 more than were currently operating according to the data used for the analysis. So, the new cap allows for more EGMs into the community venue market. Larger community venues (i.e. those already with the maximum allowable number of EGMs) were the first to install note acceptors in 2013/2014 and to increase their EGM numbers in 2016 following the change in cap. These larger venues also absorbed most of increase in user losses from 2013 to 2015, increasing 41% in clubs with 45 to 55 EGMs and hotels with 10 to 20 EGMs, and a further 27 and 32% increase for larger clubs and hotels respectively from 2015 to 2017. There was large variability in user losses between venues of varying size (EGM numbers). In 2017, the top 10 clubs (from a total of 30) accounted for 81% of total user losses, but only held 62% of EGMs in these venues, while the top 10 hotels (from 44) accounted for 58% of user losses, while only housing 35% of hotel EGMs. These larger venues also make considerably more money per EGM compared with smaller venues, with each EGM located in the top 10 hotels making on average $117,834 per year per EGM, compared with just $70,676 across all hotels, while in the top 10 clubs, user losses per EGM were $88,086, compared with $67,849 in all clubs. The higher user losses incurred in these larger venues likely reflects venue characteristics and accessibility [29]. The relationship between venue accessibility and gambling-related harm has been studied in the NT, with findings indicating that gambling risk is a function of accessibility to markets and venue effects, and that supply-side approaches to EGM regulation need to be applied [30]. Certainly, venues that are highly accessible (e.g. near or located in shopping centres, transport hubs etc.) have been shown to incur higher user losses, as well as instituting a higher prevalence of problem/moderate risk gamblers [31]. This may be reflected in the problem/moderate risk gambling estimates that showed between 2005 and 2015 EGM gamblers problem/moderate risk gambling prevalence increased (44 to 53%), while over the same time the overall number of EGM gamblers only increased marginally (4%), while the NT adult population increased 28% over the same time (see Tables 2 and 3), and EGM numbers increased by 15% from 1845 to 2454.

The analyses also showed that real user losses per EGM user have fallen 8.6% between 2005 ($3108 to $3995) and 2015 ($2841 to $3653), while the estimated number of NT adults gambling on EGMs increased 8.7%. The decrease in real user losses is predominantly due to the stagnating user losses following the smoking ban in 2010, which are reflected in decreases when analysing real user losses. The estimated increase in real user losses from problem gamblers based on the 40% Productivity Commission [2] estimate showed that on average EGM problem gamblers were losing between $30,915 and $39,748 per year in 2005, and increased 34% to between $41,504 and $53,362 in 2015. Since the EGM policy change, the combination of large increases in user losses (particularly since 2013), lower EGM participation rates, and increased prevalence of EGM problem/moderate risk gambling among EGM gamblers has very likely led to an increase in gambling-related harms from losses amongst EGM gamblers, and particularly amongst weekly and monthly EGM gamblers.

Of concern is that the changes to EGM policy in the NT were made without consultation with community, researchers, or gambling counselling services, and was premised by “improvements in harm minimisation measures and changes to legislation, both locally and nationally, have seen the gambling environment change over recent years and the initial concerns with note acceptors in clubs and hotels have been reduced” [32], yet no evidence was supplied or referenced in making this statement. In fact, the EGM policy changes were instigated after the government at the time dissolved the Licencing Commission “to cut red tape” [33] and centralised decision making power for gambling-related policy in the NT solely with the Director General of Licencing [34]. Furthermore, over the last few years the NT Government have released less data on EGMs publicly in the Director General of Licensing reports [19, 20, 28]. For example, in reports before 2014/15, user losses and the number of machines were published for every venue, while recent reports only publish total user losses by venue type, and the names of the top 10 venues in terms of user losses. This raises serious questions over the government’s role as regulator and receiver of significant taxation on EGM user losses, and their role in minimise harm from this gambling product to NT consumers.

In addition to the Productivity Commission [2] recommending a loading limit of $20 at a time into an EGM, they also recommended not increasing the number of EGMs in community venues, and that destination style gambling (i.e. casinos) be preferred for EGM gambling. The Productivity Commission report specifically drew attention to caps, and advised taking a “precautionary approach to the risks of harms from gaming machines” (2, p 14.1). Australia is unique in the world in having high accessibility to a known dangerous gambling product in EGMs. State and Territory governments across Australia have not taken on board and legislated recommendations laid out in the two Productivity Commission reports produced over the last 20 years [1, 2]. For example, only two jurisdictions, Queensland and Tasmania, have legislated to not allow LDWs, while research has clearly shown that LDWs increase the likelihood that a EGM gambler will keep playing through operand conditioning and will increase the addictive nature of the machine [12, 16].

### Limitations

A limitation of the analyses was that it was unable to unpack who was using the EGMs and where (i.e. casinos or community venues). The drop in EGM user losses in the casinos, while partially attributable to the decline in EGMs housed in the two casinos, may also reflect the preference of EGM gamblers to insert notes when loading up the EGM. However, it is impossible to know the overlap between EGM gamblers who gamble only at the casinos, only at community venues, or a combination of both. Interestingly, Australia has been debating for some years to introduce policy that makes EGM gamblers gamble using a specially design card that tracks spending and allow them to set pre-commitments on time and money spent EGM gambling [35, 36], which would allow for more nuanced analysis of EGM user losses.

Another approach to the analysis would have also been possible if EGM level data was available on user losses and noter acceptor status for each machine. A before and after analysis of user losses by machine would have unequivocally answered the question ‘has the installation of note acceptors (and increased load-up limit) on EGMs led to an increase the amount lost gambling on EGMs?’. Unfortunately, this data was not available from the NT Government, though it is hoped future data requests may be able to go down to the machine level, rather than the coarser level of venue.

The 2005 NT Gambling Prevalence Survey only screened regular gamblers (weekly excluding lotteries and raffle only gamblers) for problem gambling risk, while the 2015 survey screened all gamblers, with the former approach found to significantly under-estimate all three risk categories in the PGSI [18, 26]. Therefore, the 2005 estimates for problem and problem/moderate risk gambling were adjusted based on a “multiplying factors” derived from the 2015 survey data. The 2015 survey data was used to derive these adjustment factors, based on the percentage of problem and moderate risk gamblers excluded because of the regular gambler filter. This assumes that things were the same in 2005 as they were in 2015, yet there was a noticeable change from the 2005 to 2015 surveys in the reduction in weekly gambling across a range of activities including EGMs [18]. However, given the absence of published adjustment factors for regular gambler screening in older surveys, the 2015 NT survey is the best available source. In any case, estimates of user losses for the 2015 problem/moderate gambler estimates are unproblematic, and show an increase from 2005. In deriving the user losses per EGM, problem and problem/moderate risk gambler, an assumption was used with regards to the percentage of total user losses attributed to non-NT residents. There is no published data on this, so the estimates of 10, 20 and 30% of user losses were used and we expect that this would demonstrate the sensitivity of these estimates. Other assumptions used for this piece of analysis were from the 2010 Productivity Commission report on estimated percentage of user losses coming from problem gamblers, and EGM expenditure data from the 2005 NT survey. While there may be some variation in the user losses derived from EGM problem gamblers (depending on venue), the estimates used are the best available and provide an average.

## Conclusions

The recent regulatory changes in EGM policy in the NT have led to significant increases in EGM user losses in community venues, with the analysis providing evidence that the increase was very likely resultant on the change in EGM policy allowing note acceptors with a loading of up to $1000 into community venue EGMs. The affect was most notable in hotels and clubs which already housed the maximum number of EGMs, with these clubs having greater capacity and resources to update their EGM stock. The effects of the increased caps will likely see EGM user losses continue to rise at levels well above inflation.

Australian jurisdictions continue to ignore recommendations made by the Productivity Commission and public health gambling researchers to implement appropriate harm minimisation measures for EGMs, particularly those located in community venues. The analysis demonstrates that increased venue size (via additional EGMs), and modifications to EGM characteristics have had a significant impact on expenditure and related harms. It is therefore feasible that altering such venue and machine characteristics would likely to have a preventive effect, although that would be likely associated with a decline in net gambling revenue. That is, the analysis demonstrates that reductions in the load up limit, and/or the abolition of note acceptors, and reductions in the number of EGMs in venues is likely to reduce harm.

Reductions in EGM numbers in community venues were supported by more than 50% of NT adults in the 2015 survey [18]. The reduction in the minimum return to user to 85% for casino EGMs in 2013 was an unusual policy change, given analysis by Stevens et al. [18] showed that casinos return to user has consistently hovered around 91%, while community venues have been increasing the return to user on their EGMs from 87% in 2003/4 to 90.5% in 2015/16. However, research evidence demonstrates that EGM users have little comprehension of the ‘price’ of EGM gambling, and a reduction in the RTP means EGM gamblers lose money faster. Further, there is no legislated daily withdrawal limit on ATMs in community venues in the NT (currently it is the bank or ATM operator default). The introduction of daily withdrawals as imposed in Vitoria, for example, should be considered.

Additional harm prevention and minimisation interventions include reducing maximum bets; prohibiting LDWs; lessening accessibility through reduced operating hours of gaming rooms in venues; and mandatory use of pre-commitment options available set at low thresholds by default [37]. Given the demonstrated inability for Australian jurisdictions to identify and implement effective harm prevention and minimisation interventions, a national approach to gambling regulation in Australia may be desirable. There is no doubt that the Australian government possesses the legislative ability to impose uniform national standards.

A holistic public health approach to harm prevention and minimisation, could include transparency and consultation around gambling policy changes; data availability for consistent monitoring and evaluation; national co-ordination of research, particularly on EGMs and online betting; improved health promotion around harms associated with gambling; and ensuring services are available not only for those experiencing gambling problem personally, but for those affected by other’s gambling. The 2010 Productivity Commission report argued that “governments have improved their policy-making and regulations with respect to gambling, but significant governance flaws remain in most jurisdictions, including insufficient transparency, regulatory independence and coordination” (2, page 3). If State and Territory jurisdictions are unable to address these challenges, it is appropriate for the Australian government to do so.

## Abbreviations

CATI: Computer Aided Telephone Interviewing
CPI: Consumer Price Index
EGM: Electronic Gambling Machine
LDWs: Losses disguised as wins
NSW: New South Wales
NT: Northern Territory
PGSI: Problem Gambling Severity Index
RTP: Return to player
WHO: World Health Organization
