# Correction to: Evaluating changes in electronic gambling machine policy on user losses in an Australian jurisdiction

**DOI:** 10.1186/s12889-019-7295-y

**Published:** 2019-07-23

**Authors:** Matthew Stevens, Charles Livingstone

**Affiliations:** 1 Menzies School of Health Research, Charles Darwin University, PO Box 41096, Casuarina, NT 0811 Australia; 2 School of Public Health & Preventive Medicine, Monash University, 553 St Kilda Road, Melbourne, Vic 3004 Australia

**Correction to: BMC Public Health (2019) 19:517**

**http://orcid.org/10.1186/s12889-019-6814-1**

It was highlighted that in the original article [1] Fig. 3 and Fig. 4 legends were incorrect. This Correction article shows Fig. 3 and Fig. 4 with their correct legend.

**Fig. 3:**
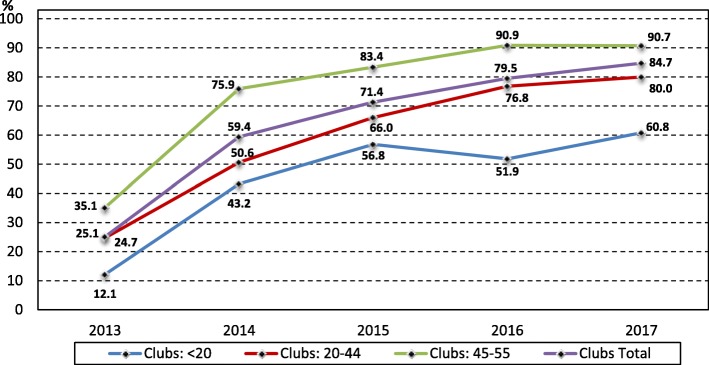
Percentage of EGMs with note acceptors in NT clubs by club size, 2013 to 2017

**Fig. 4:**
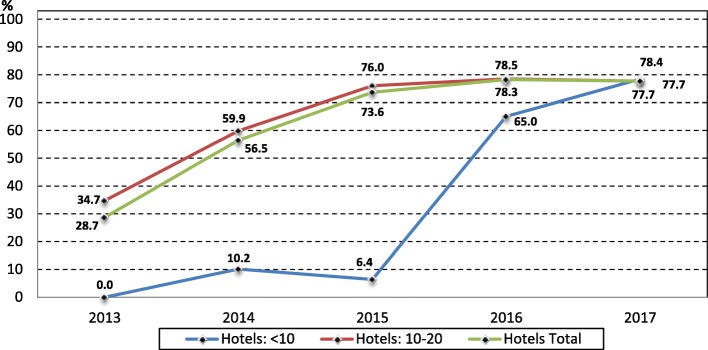
Percentage of EGMs with note acceptors in NT hotels by hotel size, 2013 to 2017
